# Optimizing Transport Carrier Free All-Polymer Solar Cells for Indoor Applications: TCAD Simulation under White LED Illumination

**DOI:** 10.3390/polym16101412

**Published:** 2024-05-16

**Authors:** Marwa S. Salem, Mohamed Okil, Ahmed Shaker, Mohamed Abouelatta, Arwa N. Aledaily, Kawther A. Al-Dhlan, Mohammad T. Alshammari, Mostafa M. Salah, Mona El Sabbagh

**Affiliations:** 1 Department of Computer Engineering, College of Computer Science and Engineering, University of Ha’il, Ha’il 55211, Saudi Arabia; ms.basyoni@uoh.edu.sa; 2 Department of Basic Engineering Sciences, Benha Faculty of Engineering, Benha University, Benha 13512, Egypt; mohamed.okil@bhit.bu.edu.eg; 3 Department of Engineering Physics and Mathematics, Faculty of Engineering, Ain Shams University, Cairo 11535, Egypt; ahmed.shaker@eng.asu.edu.eg (A.S.); mona.mohammed@eng.asu.edu.eg (M.E.S.); 4 Electronics and Electrical Communication Department, Faculty of Engineering, Ain Shams University, Cairo 11535, Egypt; m.abouelatta@eng.asu.edu.eg; 5 Department of Information and Computer Science, College of Computer Science and Engineering, University of Ha’il, Ha’il 55211, Saudi Arabia; a.aledaily@uoh.edu.sa (A.N.A.); k.aldahlan@uoh.edu.sa (K.A.A.-D.); md.alshammari@uoh.edu.sa (M.T.A.); 6 Electrical Engineering Department, Future University in Egypt, Cairo 11835, Egypt

**Keywords:** all-polymer, solar cells, indoor, LED, power conversion efficiency

## Abstract

This work inspects the utilization of all-polymer solar cells (APSCs) in indoor applications under LED illumination, with a focus on boosting efficiency through simulation-based design. The study employs a SCAPS TCAD device simulator to investigate the performance of APSCs under white LED illumination at 1000 lux, with a power density of 0.305 mW/cm^2^. Initially, the simulator is validated against experimental results obtained from a fabricated cell utilizing CD1:PBN-21 as an absorber blend and PEDOT:PSS as a hole transportation layer (HTL), where the initial measured efficiency is 16.75%. The simulation study includes an examination of both inverted and conventional cell structures. In the conventional structure, where no electron transportation layer (ETL) is present, various materials are evaluated for their suitability as the HTL. NiO emerges as the most promising HTL material, demonstrating the potential to achieve an efficiency exceeding 27%. Conversely, in the inverted configuration without an HTL, the study explores different ETL materials to engineer the band alignment at the interface. Among the materials investigated, ZnS emerges as the optimal choice, recording an efficiency of approximately 33%. In order to reveal the efficiency limitations of these devices, the interface and bulk defects are concurrently investigated. The findings of this study underscore the significance of careful material selection and structural design in optimizing the performance of APSCs for indoor applications.

## 1. Introduction

Photovoltaics are one of the most effective renewable energy sources [1]. The role of photovoltaics in energy harvesting for a low-carbon society is increasingly recognized, with a particular focus on indoor photovoltaics (IPVs) as they harness photon energy derived from household lighting or dim ambient environments [2]. 

This growing interest can be attributed to several factors, including the emergence of efficient thin-film solar cells like organic and hybrid perovskites, the transition to solid-state white light emitting diodes (LEDs) and fluorescent lamps indoors, the rise of Internet of Things (IoT) technology, and the decreasing power requirements of wireless sensors [3,4]. IPVs have the potential to power low-consumption electronic devices locally, particularly IoT sensors, which only require minimal power to operate (μW–mW) [5,6].

To efficiently harness indoor light, PV materials with relatively wide bandgaps (1.7–2 eV) and narrow absorption spectra in the visible range are essential. This ensures a good spectral alignment with the emission spectra of artificial light sources, leading to a higher open-circuit voltage (*V*_OC_) and improved IPV efficiency [7]. Among various PV materials, organic materials, in particular, have garnered significant attention for IPV applications due to their solution processing capability, flexibility, and adjustable absorption spectra [8]. Recent advancements in organic IPV have pushed the power conversion efficiency (PCE) beyond 30% and demonstrated device operation lifetimes exceeding 1000 h [9]. All-polymer solar cells (APSCs), which utilize a physical blend of *p*- and *n*-type polymers as the photoactive layer, are a subtype of organic PVs gaining traction for IPV applications, offering superior mechanical stability and morphological durability [9]. Although APSCs have been extensively researched in outdoor applications [10,11,12,13,14,15], their applications in indoor photovoltaic applications still lag. Although a few studies have demonstrated high *V*_OC_ and PCE in APSCs tailored for IPV applications, these investigations show the potential of APSCs as efficient and durable IPV solutions [16].

A blend of a polymer acceptor (*E_g_* = 1.95 eV) and a polymer donor (*E_g_* = 1.93 eV) has been utilized as the active layer, as reported in [17]. While the APSC only demonstrated an efficiency of 7.9% under AM1.5G illumination, it achieved a high PCE of 26.2% and 21.7% under FL and LED sources at 1000 lux [17]. In [18], an APSC has been fabricated with an inverted configuration of ITO/ZnO/absorber/MoO_3_/Ag, where the photoactive absorber is PM6:*b*-PYT. The solar cell delivers a PCE of 13.44% under a 1000 lux cold white LED environment. Additionally, the device showed superior thermal stability. In [19], the authors incorporated non-fused electron acceptors in APSCs, with the structure ITO/PEDOT:PSS/absorber/PFN-Br/Ag, for application under indoor environments. A high *V*_OC_ of nearly 1.0 V was achieved, accompanied by a PCE above 14% under 1500 lux and 3000 K. Moreover, an APSC module has been fabricated, with an active area of 10 cm^2^, that employs a blend of CD1 as a polymer donor CD1 and PBN-21 as a polymer acceptor. For white LED illumination, the module achieved a PCE of about 12% at 1000 lux [20]. 

Apart from this, the design and optimization of solar cell devices tailored for indoor applications hold significant importance. However, the complex interplay of factors such as low light intensity, varying spectra, and material properties necessitates sophisticated modeling and simulation techniques [21]. In this context, TCAD (Technology Computer Aided Design) device simulation emerges as a robust tool for accelerating the development and optimization of indoor solar cells [22,23]. Simulation plays a pivotal role in guiding experimental trials by offering a cost-effective and efficient means of exploring the physical properties of new materials. Before embarking on costly and time-consuming experimental inquiries, theoretical exploration through TCAD analyses provides valuable insights into the behavior of materials under various conditions. 

One of the key advantages of TCAD simulation is its ability to predict the performance of solar cells under varying operating conditions. This predictive capability allows for informed decision making during the design and optimization process. Moreover, through detailed simulations of carrier transport, recombination, and photon absorption, one can identify design parameters that enhance efficiency while minimizing material and manufacturing costs. As the APSC is a newcomer in indoor applications, very limited endeavors have been invoked in its simulation. In [24], the performance of APSCs’ tandem based on two complementary polymer blends, PM7:PIDT and PM6:PYIT, was assessed utilizing Silvaco TCAD simulations. An efficiency of about 27% for a color temperature of 2900 K was achieved under the current matching condition of the tandem configuration [24].

To attain higher PCEs in indoor settings, several design principles are recommended [25]. Firstly, it is essential to ensure that the photoactive absorber’s photo response closely aligns with the spectrum of indoor lighting, thus minimizing transparency losses. Additionally, achieving a high External Quantum Efficiency (EQE) is critical for mitigating the thermalization of photoexcited charge carriers [26]. This can be performed by the appropriate choice of polymer photoactive blend. Secondly, efforts should be made to minimize the loss of *V*_OC_. Notably, the *V*_OC_ of devices inevitably decreases due to the significant drop in light source intensity [27]. Therefore, maintaining a high *V*_OC_ under low light conditions is imperative for achieving high efficiency. This can be conducted by the appropriate design of the band alignment at the device interfaces. Thirdly, as light intensity decreases from AM1.5 to indoor conditions, there is a dramatic decline in carrier density, leading to significant trap-assisted recombination that impedes the efficient collection of charge carriers [28]. Thus, investigating the degree of defects within the bulk or at the interfaces of the solar cell under study is essential to determine the PCE limitations. 

Drawing from the preceding discourse, our study delves into the utilization of APSCs for indoor applications. The presented approach incorporates a simulation-based design using a SCAPS (Solar Cell Capacitance Simulator) device simulator, offering a comprehensive analysis of APSC performance under white LED illumination. The simulator modeling techniques are firstly justified against experimental data extracted from a fabricated cell utilizing CD1:PBN-21 as a photoactive blend and PEDOT:PSS as an HTL [20]. Through meticulous examination of both conventional and inverted cell structures, we explore suggested inorganic materials and design strategies to enhance efficiency, thereby bridging the gap between theoretical understanding and practical implementation. The study highlights the importance of maintaining high *V*_OC_ under low-light conditions to ensure optimal device performance. This is conducted by thoroughly investigating the band alignment between the relevant interfaces. Furthermore, our study conducts a thorough investigation into trap-assisted recombination mechanisms, encompassing the scrutiny of both bulk and interface defects. This meticulous examination underscores the pivotal role of defects in dictating the efficiency ceiling of APSCs. 

Overall, our simulation study offers valuable insights into the design and optimization of APSCs tailored for indoor applications. All simulations are performed under the influence of warm LED illumination at 2900 K and 1000 lux within the SCAPS simulator. 

## 2. Methods and Materials

### 2.1. SCAPS Simulation Procedure

In all simulations throughout this work, the SCAPS device simulator is used. SCAPS emerges as a pivotal tool in the development of outdoor and indoor solar cell technologies owing to its multifaceted capabilities and distinct advantages [29]. Firstly, SCAPS facilitates precise modeling of device structures and material properties tailored for both outdoor and indoor applications. By defining intricate parameters such as layer compositions, doping profiles, and interface characteristics within the simulation environment, researchers can accurately replicate the complexities inherent in various types of solar cells. This level of customization enables detailed investigation and optimization of device architectures optimized for different lighting conditions. Secondly, SCAPS integrates sophisticated optical modeling capabilities essential for simulating light absorption within a solar cell. By incorporating optical properties such as absorption coefficients and refractive indices of materials, SCAPS accurately predicts the behavior of photon absorption within the device. Furthermore, SCAPS facilitates comprehensive electrical modeling, encompassing carrier transport and recombination mechanisms crucial for solar cell performance. By incorporating parameters such as charge carrier mobility, lifetime, and recombination rates, SCAPS provides insights into the behavior of charge carriers within the device, enabling the assessment of performance-limiting factors and the identification of strategies for mitigating losses under low-light conditions. Additionally, SCAPS offers flexibility in parameter adjustment. This facilitates the simulation of various operating conditions and assesses the robustness of indoor solar cell designs [30].

**Figure 1:**
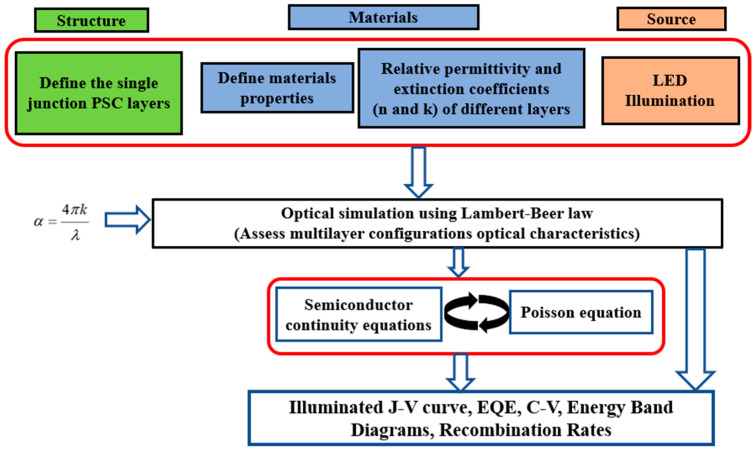
Simplified flowchart for SCAPS-1D device simulator, representing the involved input factors.

Within SCAPS, a range of physical equations are employed to elucidate the complex interplay of optical, electrical, and material properties governing device performance. The Lambert–Beer law is utilized to trace optical behavior, which describes light absorption, reflection, and transmission within the device layers, accounting for factors like material absorption coefficients and refractive indices. Electrical equations, including Poisson’s equation, the drift–diffusion equation, and continuity equations, govern carrier transportation and recombination processes (including Shockley–Read–Hall (SRH) and Auger recombination) within the device. These equations model the movement of electrons and holes under the impact of electric fields and carrier gradients, shedding light on charge carrier dynamics and recombination mechanisms. Additionally, material-specific physical quantities, such as those describing bandgap energy, doping concentration, and carrier mobilities, provide insights into the semiconductor properties essential for solar cell operation. Through the integration of these diverse physical equations and models, researchers can construct comprehensive models of solar cell structures, enabling the prediction and optimization of device performance across a wide scale of operating conditions [31]. Figure 1 presents a flowchart of the SCAPS simulator, showing the key semiconductor equations and the main required input factors.

### 2.2. Basic Structure and Layer Materials

**Figure 2:**
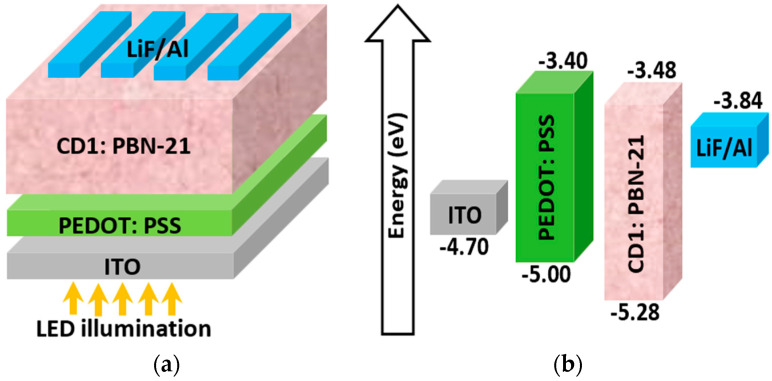
(**a**) Basic cell arrangement of the investigated APSC and (**b**) energy band edge levels before contact.

Figure 2a presents a schematic depiction of the fundamental structure of the single-junction APSC, illustrating the key stacked layers, while Figure 2b depicts the energy band levels of the different layers before contact. The structure comprises ITO/PEDOT:PSS/photoactive layer/LiF/Al. A summary of the fabrication processes is given as follows [20]. The spin-coating of the PEDOT:PSS solution onto the ITO glass substrates was performed at 5000 rpm to achieve a thickness of 40 nm. Subsequently, the active layer composed of CD1 and PBN-21 blend was fabricated via blade-coating, using a shearing rate of 35 mm/s, resulting in a thickness of 110 nm. Finally, thermal deposition was utilized to apply a 0.5 nm LiF layer followed by a 100 nm Al layer onto the photoactive layer [20]. The photoactive all-polymer material utilized is a blend of CD1 and PBN-21, featuring an optical band gap of about 1.9 eV. The thin PEDOT:PSS layer functions as a hole transportation layer. The front electrode is formed by ITO, while the back electrode is Al. To control the work function of the back contact, LiF is utilized as a very thin passivation layer [32,33]. 

Table 1 presents the parameters of the materials used in the distinct layers of the PV cell. The lowest unoccupied molecular orbital (LUMO) and the highest occupied molecular orbital (HOMO) energy levels of CD1 and PBN-21 are measured by cyclic voltammetry, while the electron and hole mobilities were determined by the space-charge limited current method [20]. Other parameters are fitted or extracted from the literature [34,35]. Additionally, Table 2 addresses the main bulk and interface defect parameters. Moreover, Table 3 shows the main factors concerning the front (ITO) and back (LiF/Al) contact materials, including electron and hole surface recombination velocities (SRV_n_ and SRV_p_) and work function (WF) values [14,32,33].

### 2.3. Calibration of APSC

**Figure 3:**
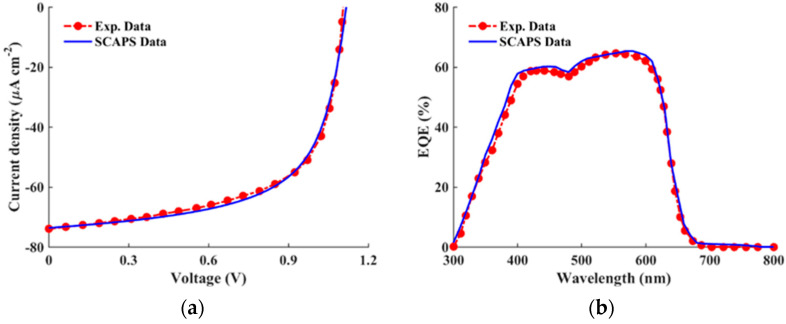
All-Polymer cell: (**a**) *J*–*V* and (**b**) *EQE* curves of simulation against experimental data [20] under LED illumination.

**Figure 4:**
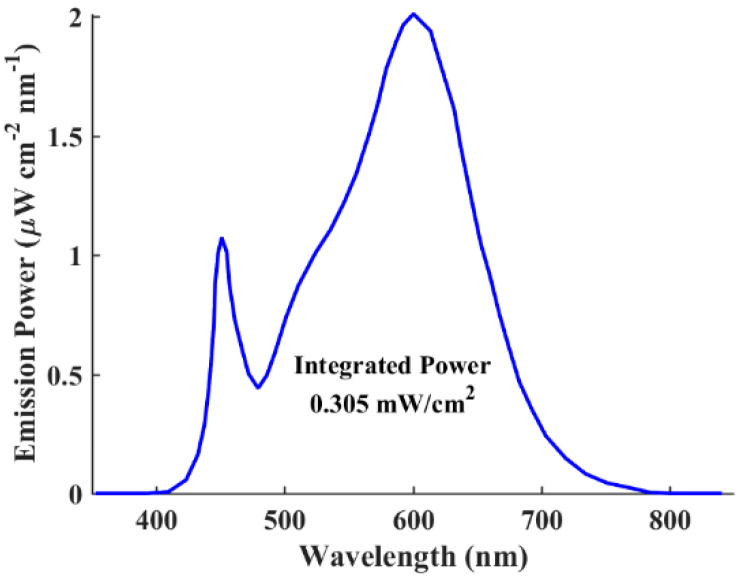
Emission power spectrum of the LED lighting source at 1000 lux with an integrated power of 0.305 mW/cm^2^ [20].

Firstly, our study involves the calibration of simulated solar cells against experimentally fabricated counterparts to validate the accuracy and reliability of our simulation model. By comparing key performance metrics such as efficiency, current–voltage characteristics, and spectral response, we assess the agreement between simulated and experimental data. Additionally, by identifying discrepancies between simulated and experimental results, we gain valuable insights into potential areas of improvement that can be invoked in our simulation approach. After applying all the material parameters and physical models discussed in the previous subsections, the illuminated J-V and EQE curves are simulated for the ITO/PEDOT:PSS/CD1:PBN-21/LiF/Al APSC structure. The simulated curves along with experimental data are shown in Figure 3a,b for the *J–V* and EQE characteristics, respectively. Warm LED illumination is utilized to examine the indoor environment’s impact on APSCs under investigation. Notably, warm LED lighting typically emits light in the lower color temperature range, usually between 2700 K to 3500 K, resulting in a more yellowish hue compared to daylight or cool white LEDs. The emission power spectrum is illustrated in Figure 4, where the integrated power of this white LED is 0.305 mW/cm^2^ (1000 lux) [20]. Moreover, the quantitative PV metrics of simulated and experimental APSCs are addressed in Table 4. 

The results displayed in Figure 3 and Table 4 signify the success of our calibration process, which is evident in the close agreement observed between the experimental and simulated data. This consistency in results validates the accuracy and reliability of our simulation model, affirming its ability to effectively capture the behavior of the practical APSC. Furthermore, Figure 4 displays the emission power spectrum of the utilized LED lighting source (1000 lux, 0.305 mW/cm^2^). It is evident from the EQE curves in Figure 3b, which are located in the visible region, and the LED spectrum in Figure 4, that the absorption spectra of the all-polymer materials align closely with the emission spectra of indoor artificial light source, indicating robust light-harvesting potential indoors.

### 2.4. Design Rules for Enhancing Cell Performance

**Figure 5:**
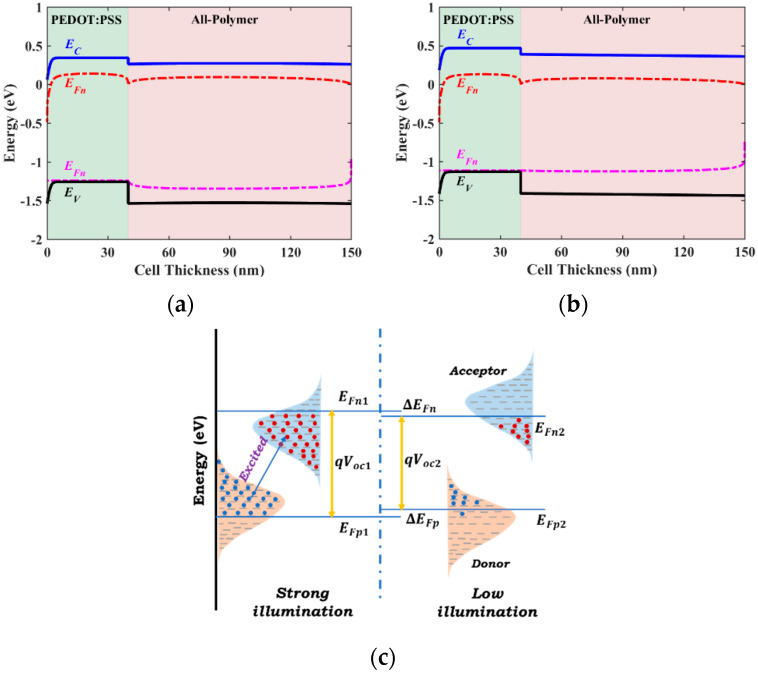
(**a**) Energy band profile at the open-circuit condition under strong illumination. (**b**) Energy band profile at the open-circuit condition under low illumination. (**c**) Quasi-Fermi levels under strong and weak illuminations.

Before delving into the simulation of the different types of APSCs, the design guidelines to optimize efficiency are discussed in this subsection. To achieve high performance in indoor solar cells, adherence to three fundamental rules is paramount [25]. Firstly, it is imperative that the photo response of the photoactive absorber closely matches the spectra of the indoor lighting sources. Thus, the proper choice of absorber material is crucial. Secondly, it is vital to suppress the loss of *V*_OC_ as far as possible. Transitioning from 1-sun radiation to indoor LED conditions inevitably leads to a decrease in *V*_OC_, attributable to the significant drop in light source intensities. To illustrate this behavior, the energy band diagrams for both strong illumination (AM1.5G, 1-sun) and low illumination (LED source) are demonstrated in Figure 5a,b. The energy band profiles for both cases are plotted at the open-circuit condition. Notably, *V*_OC_ is described by the difference in hole and electron quasi-Fermi levels. As illustrated in Figure 5c, these quasi-Fermi levels are correlated with the intensity of illumination from lighting sources. With reducing intensities, the electron quasi-Fermi level shifts upward and the hole quasi-Fermi level shifts downward, resulting in a decline in *V*_OC_. Thirdly, as the intensity decreases from AM1.5 to indoor LED illumination, the charge carrier concentration experiences a significant decline, leading to pronounced trap-assisted recombination that hampers the efficient collection of charge carriers. Under dim light intensity, traps can affect all optoelectronic metrics. Therefore, mitigating trap-assisted recombination is crucial for realizing high efficiencies for indoor APSCs. 

Understanding and addressing these factors are vital for maximizing the efficiency of indoor APSCs and advancing the field of IPV technology. As shown in Figure 3b and Figure 4, the good match between the EQE and LED spectrum proves the appropriate spectral overlap between emission and absorption, ensuring the mitigation of the thermalization of photogenerated charges. Thus, in order to design the APSC under investigation, we need to explore possible routes to boost *V*_OC_. Also, investigating the bulk and interface defects impact is imperative to determine the PCE limitation. Therefore, these design concepts will be employed, as outlined in the subsequent section.

## 3. Results and Discussion

In this section, we explore different configurations of APSCs to provide design guidelines aimed at enhancing cell performance. Remarkably, the primary objective of employing ETL and HTL is to facilitate the movement of photo-excited carriers from the absorber toward contacts in heterojunction solar cells. Additionally, both ETL and HTL serve to impede the motion of holes or electrons from the absorber to contacts, respectively, thereby minimizing the leakage current. However, the presence of interface defects at the ETL/absorber or HTL/absorber interfaces promotes electron–hole recombination, thereby deteriorating the overall cell performance. Hence, to mitigate the interface defects associated with the heterointerfaces, we opt for carrier transport layer-free designs. This approach helps to mitigate the cost and reduces the fabrication steps. Hence, two structures are under consideration: the initial ETL-free structure and an inverted HTL-free configuration.

Firstly, we delve into the investigation of the initial ETL-free structure by studying the band alignment impact between the absorber and the HTL. In this context, various materials are scrutinized for their suitability as HTLs, as detailed in Section 3.1. Advancing further, we delve into the design intricacies of the inverted HTL-free APSC in Section 3.2, emphasizing the importance of accomplishing auspicious band alignment between the absorber/ETL and ETL/contact to ease efficient charge extraction and minimize recombination losses. For this inverted configuration, we explore different ETL materials to identify the one that yields the highest PCE. 

Subsequently, Section 3.3 presents the results obtained from the best configurations identified, highlighting the significant improvements achieved. Additionally, in Section 3.4, we investigate the impact of bulk and interface defects on both optimized cells’ performance. Through a comprehensive examination of these factors, we aim to provide valuable insights and recommendations for enhancing the efficiency and functionality of APSCs in indoor applications under the influence of white LED illumination.

### 3.1. Impact of Band Alignment in ETL-Free APSC

By offering a favorable energy level alignment with the valence band of the active layer material, the HTL facilitates the smooth flow of holes toward the electrode, thereby minimizing charge recombination losses. However, to ensure a continuous and unhindered flow of charge carriers to the electrode, it is essential to establish an appropriate band alignment between the contact and the HTL.

Furthermore, the choice of HTL material and its properties, such as hole mobility and conductivity, significantly influence the charge transport dynamics within the device. Optimal selection and engineering of HTL materials can lead to enhanced carrier transport, thereby reducing recombination losses. Organic HTL materials are commonly utilized; however, they suffer from weak stability and low hole mobility [36]. This instability arises from the morphological changes that organic materials undergo under thermal conditions, leading to alterations in their properties over time. One effective strategy to address the instability of organic HTLs involves substituting these organic materials with inorganic alternatives, which typically offer enhanced stability and reliability [37]. Moreover, regarding cost aspects, unlike organic HTL materials like PEDOT:PSS, priced at 200–250 USD/gram, inorganic HTL materials typically range from 10–15 USD/gram or less [38]. This indicates their supportive role in reducing the cost of APSCs.

**Figure 6:**
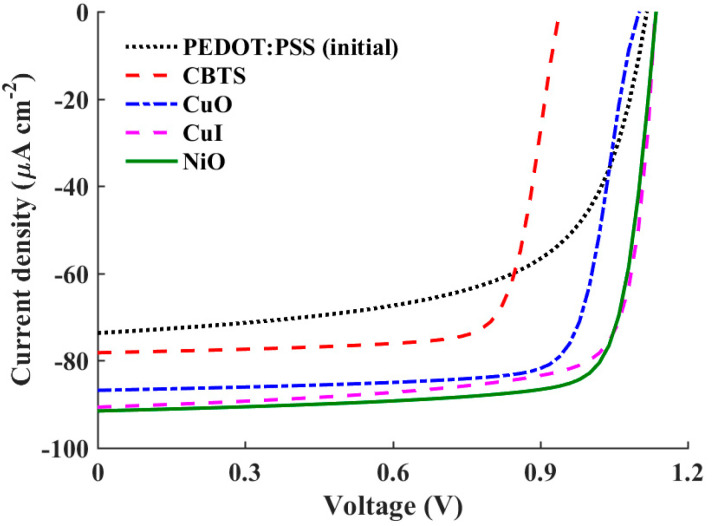
*J–V* curves of a conventional APSC with distinct HTL materials.

Thus, in this simulation run, four inorganic HTL materials are examined for potential utilization in the APSC under consideration. Table 5 provides an overview of the various input technological parameters associated with these materials. Additionally, Figure 6 illustrates the simulated *J-V* characteristic curves of these materials alongside the initial structure, which utilizes the organic material PEDOT:PSS as an HTL. The quantitative results of this simulation are given in Table 6, where the output optoelectronic metrics of all materials are listed. Although the initial cell exhibits a competitive *V*_OC_, its short-circuit current (*J*_SC_) is notably the lowest among all tested HTL materials. Conversely, it is evident that NiO demonstrates the most impressive performance, boasting the highest *V*_OC_ and *J*_SC_ values, consequently achieving the maximum PCE of 27.19%. Notably, CuO also delivers a high PCE of 24.38%; however, the *J-V* characteristics reveal an S-curve, which may be attributed to interface recombination or non-ideal charge transport phenomena. While this kink effect is relatively minor, it remains undesirable. Additionally, it is noteworthy that CuI also demonstrates commendable performance, achieving a PCE of 26.22%, slightly lower than that of NiO. 

To elucidate the distinctions among the studied HTL materials, two crucial parameters, namely the valence band offset (VBO) at the polymer/HTL interface and the barrier height at the contact side (*φ_p_*), require careful investigation. These values are provided in Table 6 for the HTL materials under study. The VBO holds significant importance in shaping the performance of heterojunction solar cells. A negative VBO signifies the formation of a cliff-like energy barrier at the HTL/absorber interface. If the value of the negative VBO is relatively high, the interface acts as a substantial impediment to the efficient extraction and transport of charge carriers, leading to an increased rate of electron–hole recombination. Consequently, key parameters such as the *V*_OC_ experience a substantial decline. The high negative VBO thereby undermines device performance by impeding the smooth flow of charge carriers across the interface [41]. Conversely, a low positive VBO indicates a more favorable interface configuration. In such instances, the energy level alignment between the HTL and the absorber material facilitates efficient charge extraction and transport [42]. However, if the barrier height is relatively high, the hole flow will not be smooth toward the contact side. Thus, a compromise between the VBO and *φ_p_* is essential to ensure optimal carrier transport. 

In the case of the initial cell employing PEDOT:PSS as the HTL, although *φ_p_* is relatively low, the VBO is highly negative (−0.28 eV). This combination results in increased recombination losses, which are manifested in lower values of the *V*_OC_, FF, and, ultimately, the lowest attainable PCE. Conversely, for CuI, the VBO is slightly negative, which positively influences the *V*_OC_, FF, and PCE. In contrast, NiO demonstrates a significantly lower VBO value of −0.02 eV, effectively reducing recombination losses and thereby enhancing overall device performance. This suggests that NiO serves as a more effective HTL material in mitigating recombination losses compared to the other materials. However, in the case of CuO, while the VBO is slightly positive, the resultant increase in barrier height impedes hole transport, leading to a deterioration in device performance. Furthermore, the performance of the solar cell deteriorates significantly for CBTS (Copper-Barium-Thiostannate), primarily due to the relatively high positive VBO and *φ_p_* of 0.8 eV compared to the other studied HTL materials. This unfavorable combination significantly hampers charge transport and extraction efficiency, resulting in a decrease in the PCE. 

**Figure 7:**
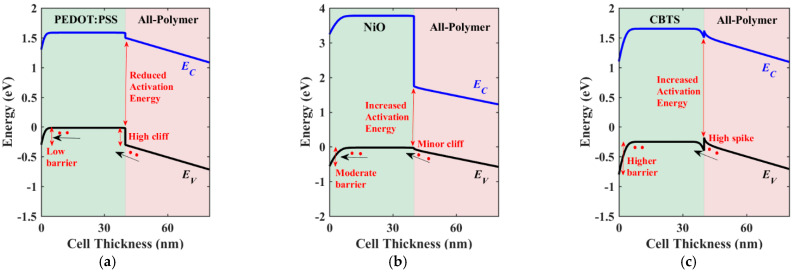
Illuminated energy band diagrams at the short-circuit condition of conventional APSC with different HTL materials: (**a**) PEDOT:PSS, (**b**) NiO, and (**c**) CBTS.

The energy band profiles for three HTL materials are displayed in Figure 7. The profiles illustrate the VBO and *φ_p_* and their roles in carrier transportation for PEDOT:PSS (Figure 7a), NiO (Figure 7b), and CBTS (Figure 7c). In Figure 7a, the movement of holes is hindered by the high cliff formed at the absorber/HTL interface, despite encountering a low barrier at the contact side. Additionally, this high cliff results in a reduced activation energy, which increases interfacial recombination. Moreover, there is a possibility of electron leakage current due to the low barrier faced from the PEDOT:PSS side. Conversely, in Figure 7b, hole transport is facilitated across the interface toward the contact. This is attributed to the low cliff at the interface as well as the moderate barrier height at the contact, allowing for smoother hole flow. Furthermore, the activation energy is increased, indicating lower recombination. Moreover, any leakage currents are minimized due to the barrier encountered by the electrons. Finally, in Figure 7c, a high spike is observed. While this spike does not impede hole flow, the high barrier encountered at the contact poses an issue. Additionally, the low barrier faced by the electrons may lead to undesirable leakage currents.

### 3.2. Impact of Band Alignment in Inverted HTL-Free APSC

**Figure 8:**
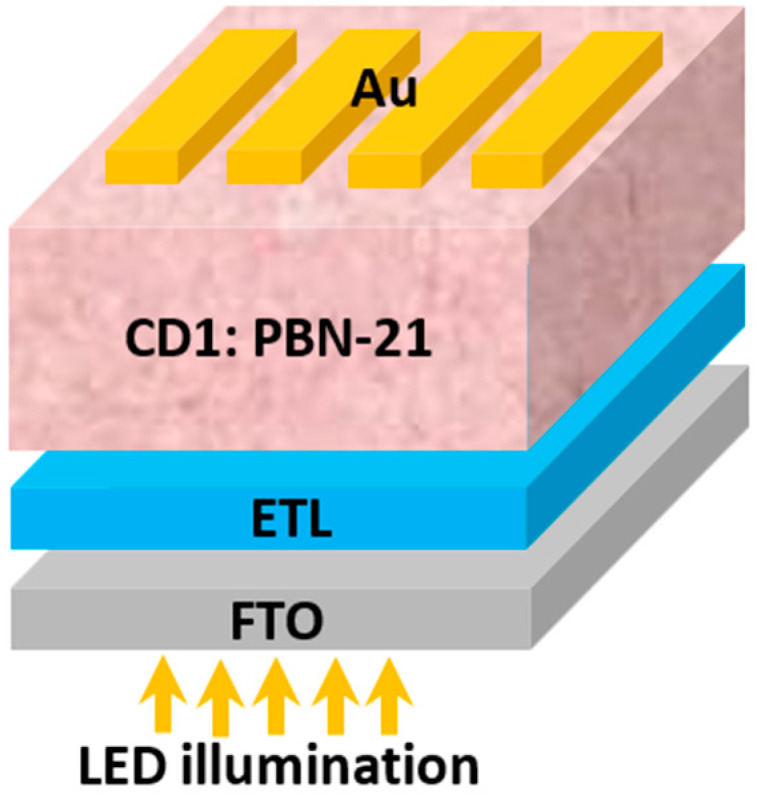
Device design of the proposed inverted structure without hole transport material.

Here, an inverted APSC architecture is proposed and investigated. The inverted structure comprises an ETL to face the illumination, instead of the HTL. The inverted HTL-free structure is shown in Figure 8. In this structure, the contacts are modified, with FTO and Au serving as the front and back contacts, respectively. The work functions of these contacts are determined to be 4 eV and 5.1 eV, respectively [43]. As shown in the figure, the simulated inverted structure comprises the stacked layers FTO/ETL/absorber/Au. The design of such a structure is based on establishing favorable energy level alignment with the conduction band of the active layer material and the ETL. This will ensure a smooth flow of electrons towards the electrode, thereby minimizing recombination rates. Furthermore, the establishment of an appropriate band alignment between the ETL and the electrode is essential to maintain a continuous and unimpeded flow of charge carriers toward the electrode. Additionally, the selection and engineering of ETL materials and their properties, such as electron mobility and conductivity, significantly impact the charge transport dynamics within the device [30,43]. By optimizing the choice of ETL materials, it is possible to enhance electron transport efficiency and minimize recombination losses, ultimately leading to improved device performance and higher solar cell efficiency. Specifically, most inorganic ETL materials offer promising high physicochemical stability under various environmental conditions besides their simple preparation process, making them attractive candidates for long-term device reliability. Furthermore, compared to the traditional organic PCBM ETL, which costs USD 1000 per gram, inorganic ETL materials are typically estimated to range from USD 0.1 to USD 1 per gram [44].

**Figure 9:**
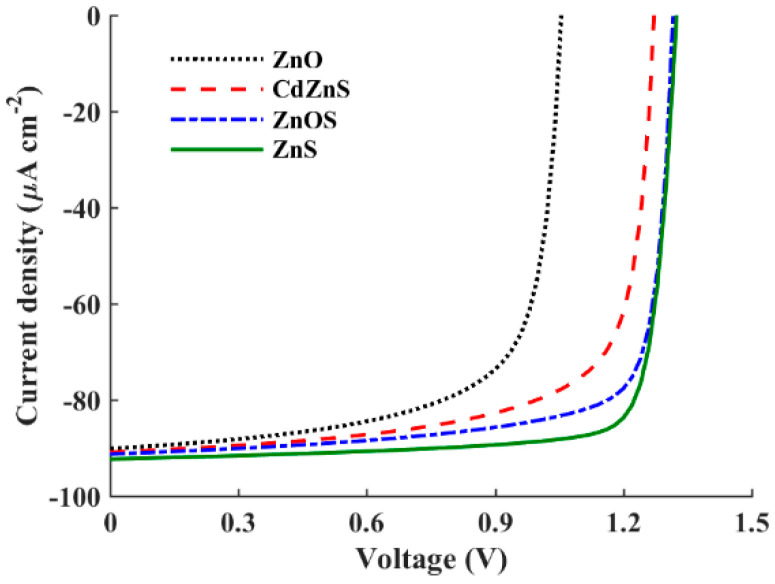
*J–V* curves of an inverted APSC with different ETL materials.

In the following simulation endeavor, we scrutinize four different inorganic ETL materials for their potential integration into the inverted APSC structure. A comprehensive overview of the various input technological parameters associated with these ETL materials is presented in Table 7. Moreover, Figure 9 provides a visual representation of the simulated J-V characteristic curves of these materials. Quantitative results derived from this simulation are detailed in Table 8, encompassing the output optoelectronic metrics for all materials under scrutiny. As evidenced by the results, ZnS emerges as the top performer among the materials tested, boasting a PCE of 33.02%. Following closely behind are the ZnOS and CdZnS ternary compounds, which achieve PCEs of 30.64% and 27.18%, respectively. On the other hand, ZnO exhibits the lowest PCE among the tested materials, recording a value of 21.75%.

To delineate the differences among the studied ETL materials, two critical parameters, namely the conduction band offset (CBO) at the absorber/ETL interface and the barrier height at the contact side (*φ_n_*), necessitate thorough examination. The corresponding values regarding our investigated device are presented in Table 8 for the studied ETL materials. The CBO holds paramount importance in shaping the performance of heterojunction solar cells. A negative CBO occurs when the conduction band edge energy of the ETL lies below that of the absorber layer, leading to the formation of an energy cliff at the absorber/ETL interface. The energy cliff does not impede the motion of photo-generated electrons but results in increased interface recombination. The activation energy for carrier recombination is lesser than the band gap of the absorber, indicating a higher likelihood of interface recombination and subsequent reduction in *V*_OC_. Moreover, if the magnitude of the negative CBO is significant, the interface poses a substantial obstacle to the efficient transport of electrons, thereby increasing the rate of electron–hole recombination. The high negative CBO thus undermines device performance by impeding the smooth flow of charge carriers across the interface [48]. Conversely, a positive CBO forms an energy spike at the interface, which acts as a barrier to electron transport. While a spike may impede electron transport, the activation energy is increased in this case, leading to reduced interface carrier recombination. A low positive CBO suggests a more favorable interface configuration. In such cases, the energy level alignment between the absorber material and the ETL facilitates efficient charge extraction and transport. The previous literature suggests that a flat band or a slight spike-like band offset (<+0.3 eV) is more advantageous for thin-film solar cell operation when assuming ideal flat band conditions at the contact sides [49]. However, for more practical simulations, if the barrier height at the contact side, *φ_n_*, is relatively high, the electron flow may encounter obstacles. Hence, achieving a balance between the CBO and *φ_n_* is crucial to ensure optimal carrier transport.

Referring to Table 8, it is evident that ZnS exhibits the best performance among the investigated ETL materials. This superior performance can be attributed to its relatively low positive CBO value (0.04 eV), which facilitates favorable band alignment at the ETL/absorber interface, thereby reducing recombination losses. The high values of *V*_OC_ and FF observed for ZnS further corroborate the beneficial impact of this low positive CBO. Despite having the highest barrier height, *φ_n_*, among all cases, the dominant influence of the CBO on performance is evident. Conversely, the ternary compounds ZnOS and CdZnS, with lower *φ_n_* values, exhibit negative CBOs, leading to increased recombination losses and consequently lower PCEs. For ZnO, despite having the lowest *φ_n_* value, the performance deteriorates due to its high positive CBO. These results highlight the crucial role of the CBO in determining device performance, even in the presence of varying barrier heights. 

**Figure 10:**
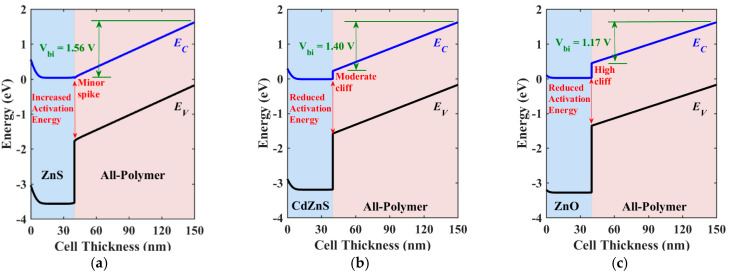
Illuminated energy band diagrams at the short-circuit condition of inverted APSC with different ETL materials: (**a**) ZnS (V_bi_ = 1.56 V), (**b**) CdZnS (V_bi_ = 1.40 V), and (**c**) ZnO (V_bi_ = 1.17 V).

Next, the energy band diagrams for three ETL materials are depicted in Figure 10. Figure 10a illustrates the band profile for the optimal ETL material, ZnS. In this case, the transfer of electrons is facilitated due to the low spike encountered at the interface, despite facing a relatively high barrier at the contact side. Additionally, this low spike results in an increased activation energy, which decreases interfacial recombination. The built-in potential, indicated in the figure, shows a high value of about 1.56 V. This built-in voltage clearly explains the high *V*_OC_ obtained in ZnS case. On the other hand, in Figure 10b, electron transport is partially hindered due to the moderate cliff formed at the CdZnS/absorber interface, which also reduces the activation energy, leading to escalated recombination. A reduced built-in potential is also observed, as shown in the figure. Finally, in Figure 10c, a high cliff is noted for the ZnO case. This cliff significantly impedes electron flow, despite the low barrier height at the contact side. Additionally, the activation energy is reduced, leading to higher recombination than in the case of CdZnS. The built-in potential in ZnO is just 1.17 V. For all cases, it can be noted that the hole leakage current is suppressed thanks to the high barrier faced by holes at the ETL side.

### 3.3. Comparison between ETL-Free and HTL-Free Structures

**Figure 11:**
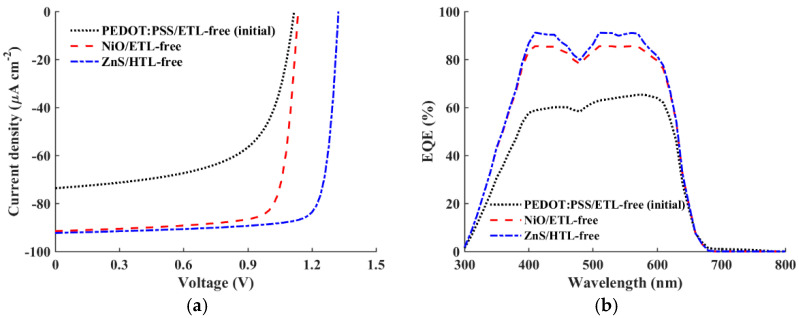
Comparison of the initial calibrated cell, best conventional cell (NiO/ETL-free), and best-inverted cell (ZnS/HTL-free): (**a**) the *J–V* characteristics and (**b**) quantum efficiency spectra.

**Figure 12:**
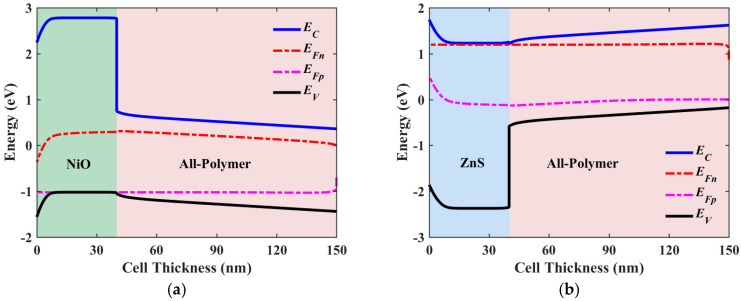
Illuminated energy band diagrams at MPP of (**a**) the optimized conventional cell (NiO/ETL-free) and (**b**) the optimized inverted cell (ZnS/HTL-free).

This subsection provides a comparison of the two optimized structures, ETL-free and HTL-free, alongside the initial configuration. The objective is to determine the device structure with the greatest potential for improving solar cell efficiency, particularly in terms of *V*_OC_. Figure 11 demonstrates the J-V (Figure 11a) and EQE (Figure 11b) characteristics. As can be inferred from the figures, the case of ZnS/HTL-free yields the highest *V*_OC_ and *J*_SC_. Despite the slightly lower *J*_SC_ observed in the NiO/ETL-free case, its *V*_OC_ is considerably lower. Comparing these results to those of the initial cell, the optimized ETL-free design exhibits a modest improvement in *V*_OC_ and a substantial enhancement in *J*_SC_. On the other hand, the optimized inverted HTL-free design demonstrates enhancements in both *V*_OC_ and *J*_SC_, suggesting the potential of HTL-free configurations for this type of APSC. Table 9 summarizes the quantitative results of the PV metrics for the three cases. As indicated in the table, the optimized HTL-free structure achieves the greatest PCE of 33.02%, with a percentage enhancement ratio of 97.13% over the initial structure. Moreover, the obtained *V*_OC_ exceeds 1.3 V, indicating a robust solution for mitigating *V*_OC_ decline under indoor illumination conditions. Meanwhile, the optimized ETL-free design achieves a lower *V*_OC_ and a lower percentage enhancement ratio. Finally, Figure 12 displays the illuminated energy band diagrams at the maximum power point MPP for the two optimized cases, the conventional cell, NiO/ETL-free (Figure 12a), and the inverted cell, ZnS/HTL-free (Figure 12b).

### 3.4. Impact of Bulk and Interface Defects on Optimized APSCs

**Figure 13:**
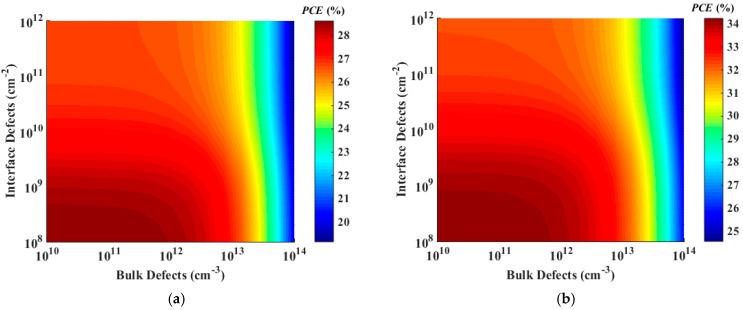
Contour graphs of PCE based on the impact of bulk and interface trap density of (**a**) the best conventional cell (NiO/ETL-free) and (**b**) the best-inverted cell (ZnS/HTL-free).

In this section, we delve into the influence of both interface and bulk defects on the performance of the two optimized ETL-free and HTL-free APSCs. This investigation is pivotal in addressing the efficiency limitations of these devices. We manipulate the density of bulk trap defects within the all-polymer layer, ranging from 11 × 10^10^ to 1 × 10^14^ cm^−3^. Simultaneously, we adjust the interfacial defects, varying from 1 × 10^8^ to 1 × 10^12^ cm^−2^, at the HTL/absorber and ETL/absorber interfaces, for the optimized ETL-free and HTL-free structures, respectively. It is worth noting that the initial values of the bulk and interface defects are 1 × 10^12^ cm^−3^ and 1 × 10^10^ cm^−2^, respectively, as indicated in Table 2. Figure 13a depicts the impact of these variations on the PCE for the ETL-free cell, while Figure 13b illustrates the effect for the HTL-free device. Notably, both devices exhibit similar behaviors. Maximum efficiencies of approximately 29% and 34% are attainable for the ETL-free and HTL-free APSCs, respectively. Furthermore, when the bulk defects are sufficiently low (ranging from 1 × 10^10^ to 1 × 10^12^ cm^−3^), the PCE reaches its peak as long as the interface defects remain at a low density of 1 × 10^9^ cm^−2^. Even with an increase in interface defects up to 1 × 10^12^ cm^−2^, the cells still demonstrate considerable performance. For instance, within the range of 1 × 10^12^ cm^−2^ interface defects, efficiencies of up to 26% and 31% can be achieved for the ETL-free and HTL-free cells, respectively. Moreover, as the bulk defects surpass 1 × 10^13^ cm^−3^, the performance of both cells declines irrespective of the interface defect density. This suggests that the impact of absorber defects is more significant than that of the interface defects for these devices.

## 4. Conclusions

Based on the exploration of various configurations and design considerations for APSCs in indoor applications, several key findings and conclusions can be drawn. Firstly, our investigation underscores the crucial role of ETL and HTL in facilitating efficient charge extraction while minimizing leakage currents. However, the presence of interface defects at the ETL/absorber or HTL/absorber interfaces can lead to electron–hole recombination, thus impairing cell performance. To address this challenge, carrier transport layer-free designs are proposed, offering a cost-effective and simplified fabrication approach. In the examination of the initial ETL-free structure, we identified various materials suitable for HTLs, aiming to optimize band alignment and minimize recombination losses. Similarly, for the inverted HTL-free configuration, different ETL materials were explored to achieve favorable band alignment and enhance charge extraction efficiency.

Through comprehensive simulations and analysis, significant improvements in cell performance were observed for the optimized configurations. The conventional ETL-free structure exhibited a PCE of approximately 27% when utilizing NiO as the HTL material. Meanwhile, the inverted HTL-free configuration demonstrated the highest potential, achieving a remarkable PCE of around 33% with ZnS as the ETL material. Furthermore, our investigation into the impact of bulk and interface defects revealed efficiency ceilings above 29% and 34% for the ETL-free and HTL-free configurations, respectively.

Overall, our study highlights the importance of meticulous design and material selection in enhancing the efficiency and functionality of APSCs for indoor applications under white LED illumination. By optimizing structural and technological parameters, significant performance improvements can be achieved, paving the way for the widespread adoption of carrier transport free APSCs in indoor energy harvesting applications. Our future work will involve not only studying the fabrication of these all-polymer/inorganic ETL or all-polymer/inorganic HTL systems but also assessing their stability under practical conditions.

## Figures and Tables

**Table 1:** Main material and technological factors of various layers of APSC.

Parameters	PEDOT:PSS	CD1:PBN-21
Thickness, *t* (nm)	40	110
*E*_LUMO_ (eV)	−3.40	−3.48
*E*_HOMO_ (eV)	−5.00	−5.28
Relative permittivity, *ε_r_*	3.0	3.0
Electron/Hole mobility, (*μ*_n_/*μ*_p_) (cm^2^/Vs)	5.0 × 10^−4^ /5.0 × 10^−4^	2.77 × 10^−4^ /4.46 × 10^−4^
Effective DOS in CB, *N*_c_ (cm^−3^)	2.2 × 10^18^	1.0 × 10^19^
Effective DOS in VB, *N*_v_ (cm^−3^)	1.8 × 10^19^	1.0 × 10^19^
Donor level, *N*_D_ (cm^−3^)	-	-
Acceptor level, *N*_A_ (cm^−3^)	1.0 × 10^19^	-
Reference	[34,35]	[20]

**Table 2:** Bulk and interface defect parameters of APSC.

Parameters	Bulk Defects		Interface Defects
	CD1:PBN-21	PEDOT:PSS	HTL/Polymer
Defect type	Single Acceptor	Single Acceptor	Acceptor
Electron/Hole capture cross-section (cm^2^)	1 × 10^−15^ /1 × 10^−15^	1 × 10^−15^ /1 × 10^−15^	1 × 10^−15^ /1 × 10^−15^
Trap energy level above *E*_v_ (eV)	0.6	0.6	0.6
Concentration (cm^−3^)	1 × 10^12^	1 × 10^14^	1 × 10^10^

**Table 3:** Main parameters of the front and rear metal contacts for APSC.

Contact	Material	WF (eV)	SRV_n_ (cm/s)	SRV_p_ (cm/s)
Front	ITO	4.70	1 × 10^7^	1 × 10^6^
Rear	LiF/Al	3.84	1 × 10^6^	1 × 10^7^

**Table 4:** PV metrics of simulated and experimental all-polymer solar cells.

PV Parameters	*V*_OC_ (V)	*J*_SC_ (µA/cm^2^)	*FF* (%)	*PCE* (%)
Experimental [20]	1.10	73.90	62.80	16.75
This work	1.11	73.67	62.03	16.75

**Table 5:** Basic factors of various inorganic HTL materials.

Parameters	NiO	CuI	CuO	CBTS
*t* (nm)	40	40	40	40
*E_g_* (eV)	3.8	3.1	2.1	1.9
*χ* (eV)	1.46	2.1	3.2	3.6
*VBO* (eV)	−0.02	−0.08	0.02	0.22
*φ_p_* (eV)	0.56	0.5	0.6	0.8
*ε_r_*	10.7	6.5	7.11	5.4
*μ_n_*/*μ_p_* (cm^2^/Vs)	12/2.8	100/43.9	3.4/3.4	30/10
*N_c_*/*N_v_* (cm^−3^)	2.8 × 10^19^ /1 × 10^19^	2.8 × 10^19^ /1 × 10^19^	2.2 × 10^18^ /1.8 × 10^18^	2.2 × 10^18^ /1.8 × 10^19^
Reference	[30,39]	[30,39]	[13,40]	[13]

**Table 6:** Band alignment and optoelectronic parameters for the conventional APSC for various HTL materials.

HTL	*φ_p_* (eV)	VBO (eV)	*V*_OC_ (V)	*J*_SC_ (µA/cm^2^)	*FF* (%)	*PCE* (%)
PEDOT:PSS	0.30	−0.28	1.11	73.67	62.03	16.75
CuI	0.50	−0.08	1.13	90.65	77.72	26.22
NiO	0.56	−0.02	1.14	91.53	79.73	27.19
CuO	0.60	0.02	1.10	86.80	77.72	24.38
CBTS	0.80	0.22	0.94	78.18	77.37	18.64

**Table 7:** Fundamental input parameters of various ETL materials.

Parameters	ZnS	ZnOS	CdZnS	ZnO
*t* (nm)	40	40	40	40
*E_g_* (eV)	3.6	2.83	3.18	3.3
*χ* (eV)	3.44	3.6	3.71	3.9
*ε_r_*	9	9	10	9
*μ_n_*/*μ_p_* (cm^2^/Vs)	100/25	100/25	340/50	50/5
*N_c_*/*N_v_* (cm^−3^)	1.8 × 10^19^ /2.4 × 10^19^	2.2 × 10^18^ /1.8 × 10^18^	2.5 × 10^18^ /2.5 × 10^19^	1 × 10^19^ /1 × 10^19^
Reference	[45]	[40]	[46]	[47]

**Table 8:** Band and PV parameters for the inverted all-polymer cell with various inorganic ETL materials.

ETL	*φ_n_* (eV)	CBO (eV)	*V*_OC_ (V)	*J*_SC_ (µA/cm^2^)	*FF* (%)	*PCE* (%)
ZnS	0.56	0.04	1.32	92.32	82.34	33.02
ZnOS	0.40	−0.12	1.31	91.27	77.72	30.64
CdZnS	0.29	−0.23	1.27	90.91	71.62	27.18
ZnO	0.10	−0.42	1.05	90.16	69.68	21.75

**Table 9:** Comparison relating initial calibrated cell, best conventional cell (NiO/ETL-free), and best-inverted cell (ZnS/HTL-free).

Cell Configuration	*V*_OC_ (V)	*J*_SC_ (µA/cm^2^)	*FF* (%)	*PCE* (%)	*Enhancement Ratio* (%)
PEDOT:PSS/absorber/ETL-free	1.11	73.67	62.03	16.75	-
NiO/absorber/ETL-free	1.14	91.53	79.73	27.19	62.33
ZnS/absorber/HTL-free	1.32	92.32	82.34	33.02	97.13

## Data Availability

No new data were created or analyzed in this study. Data sharing does not apply to this article.
